# Use of Smartphone Apps for Improving Physical Function Capacity in Cardiac Patient Rehabilitation: Systematic Review

**DOI:** 10.2196/21906

**Published:** 2021-09-17

**Authors:** Katherine Tuttle, Arpad Kelemen, Yulan Liang

**Affiliations:** 1 Department of Organizational Systems and Adult Health University of Maryland, Baltimore Baltimore, MD United States; 2 Department of Family and Community Health University of Maryland, Baltimore Baltimore, MD United States

**Keywords:** cardiac rehabilitation, physical capacity, exercise, smartphone apps

## Abstract

**Background:**

Cardiac rehabilitation (CR) is an evidence-based approach for preventing secondary cardiac events. Smartphone apps are starting to be used in CR to give patients real-time feedback on their health, connect them remotely with their medical team, and allow them to perform their rehabilitation at home. The use of smartphone apps is becoming omnipresent and has real potential in impacting patients in need of CR.

**Objective:**

This paper provides critical examinations and summaries of existing research studies with an in-depth analysis of not only the individual studies but also the larger patterns that have emerged with smartphone apps in CR as well as their significance for practice change.

**Methods:**

A systematic review was conducted through broad database searches that focused on evaluating randomized controlled trials, in compliance with the PRISMA (Preferred Reporting Items for Systematic Reviews and Meta-Analyses) expectations. A total of 43 articles were evaluated, and 6 were chosen for this review. The dates of the articles ranged from 2014-2020, and the studies focused on the population of cardiac outpatients who needed CR after suffering a cardiac event, with interventions using a smartphone that incorporated the CR standards of the American Heart Association. The outcomes measured were directed at focusing on improved exercise function capacity, valued at a significance level of *P*<.05, for improved 6-minute walk test (6MWT) and peak oxygen uptake (PVO_2_) results.

**Results:**

In the evaluated articles, the results were inconsistent for significant positive effects of CR smartphone apps on cardiac patients’ physical function capacity in terms of the 6MWT and PVO_2_ when using a smartphone app to aid in CR.

**Conclusions:**

Because evidence in the literature suggests nonhomogeneous results for successful use of smartphone apps in CR, it is crucial to investigate the potential reasons for this inconsistency. An important observation from this systematic review is that smartphone apps used in CR have better clinical outcomes related to physical function capacity if the app automatically records information or provides real-time feedback to participants about their progress, compared to apps that only educate and encourage use while requiring the participant to manually log their CR activities. Additional factors to consider during these studies include the starting health of the patients, the sample sizes, and the specific components of CR that the smartphone apps are using. Overall, more clinical trials are needed that implement smartphone apps with these factors in mind, while placing stronger emphasis on using biosensing capabilities that can automatically log results and send them to providers on a real-time dashboard.

## Introduction

Heart disease is still the leading cause of death in the United States; however, as medicine improves, survival rates for sudden and chronic heart complications are increasing, as indicated by a 34% drop in mortality rates from 2005-2015 and a predicted 27% further decline by 2030 [1]. There is now an increased need to manage these heart diseases in the long term [2]. However, we are now faced with the problem of high hospitalization reoccurrences of around 18% to 30%, which increases hospital expenses and the likelihood of mortality for patients [3]. Cardiac rehabilitation (CR) is a well-studied evidence-based secondary prevention method that has been found to decrease cardiac-related deaths by at least 26% for patients who have encountered a cardiac event, including surgery, coronary artery disease, myocardial infarction, and chronic heart disease [4,5].

There are several phases of CR, and depending on the hospital or clinic at which CR is initiated, its guidelines and definitions vary slightly. For the purpose of this review, it is stated that a full CR program typically lasts 3-8 months, depending on patient-specific goals [6]. The breakdown is as follows: Phase I of CR is considered the in-patient phase. This phase is entered after a cardiac event occurs, and it involves strengthening activities of daily living with therapists [7]. In Phase II of CR, the patient begins outpatient rehabilitation and develops a comprehensive treatment plan with health care providers; this plan often involves exercise and lifestyle modification, and it lasts approximately 3 to 6 weeks. This is crucial in the prevention of further cardiac events [7]. Phase III is the maintenance phase, where patients can decide to continue CR on their own; however, this phase is not required, nor does it have notable incremental benefits compared to Phase II [8,9].

Since 2016, it has been reported that even for eligible CR participants who were covered by Medicare, only 20%-25% used the service, and only 26% of those followed the rehabilitation program to completion [10].

In 2017, more than 250,000 patients were eligible for CR in the United States; however, less than 30% used the resource [4]. This is deemed unacceptable by the American Heart Association (AHA) [4]. Despite clinical trials and research that indicates CR programs are helpful in decreasing the occurrence of secondary coronary events, due to the patient-focused limitations of difficulty obtaining transportation to CR centers, lack of time, geographical barriers, and inability to drive, the participation in these programs is generally low [11-13].

The option of home-based focused CR has been discussed at length since 1995, with successful studies using the MULTIFIT program and the Healthy Heart Program; the AHA and the American Association of Cardiovascular and Pulmonary Rehabilitation (AACVPR) assert that home-based CR is an equivalent option to in-person CR [4]. However, in the past, home-based CR has been difficult to implement because of the many different components to address and the limited number of physicians and nurses who can be physically present to conduct it. With known cardiac event prevention through CR, a goal was established by the Million Hearts Cardiac Rehabilitation Collaborative, comprising more than 100 organizations, to increase program participation of eligible CR patients to 70% from 2016 to 2022 because it is estimated that a million cardiac events could be prevented and save 25,000 lives in the United States alone [10].

Recently, technology and health care have reached an intersection. With increased communication and research between informatics and medicine, technology will be leveraged to support the American health care system and provide flexibility to patients for CR to combat problems such as geographical barriers and transportation. Studies are showing that smartphone apps can facilitate a higher volume of patients and can be used to better manage heart conditions at home, as communication is web-based.

A myriad of components of CR are outlined by the AHA and the AACVPR that are specific to CR in the United States; these include education on nutrition with diet modification guidelines, such as sodium restriction and lipid management using fasting lipid measurements; psychosocial support; hypertension treatment through exercise; smoking cessation; diabetes management; and exercise training [14]. With the expansion of technology, many of these CR components can now be managed through a smartphone app, which allows for remote monitoring, increased completion of CR, and better clinical outcomes.

One of the most influential components for preventing secondary heart-associated problems is physical activity [11]. Therefore, exercise capacity is the focal outcome addressed and can be measured through the 6-minute walk test (6MWT) and/or peak oxygen uptake (PVO_2_). The 6MWT is a standardized way of measuring walking distance to determine exercise ability and capacity [3,11,15-17], and PVO_2_ indicates exercise capacity through anaerobic respiration measurements during exercise [18]. Furthermore, with the rapid expansion of smartphone apps, the possibility of using them with home CR or alongside traditional CR is being explored.

Many randomized controlled trials (RCTs) have evaluated the use of smartphone apps in aiding compliance with CR programs, either in a traditional center or at home; however, not many have focused on examining clinical outcomes for patients who use apps in conjunction with home or traditional center–based CR [11]. The aim of this paper is to evaluate if smartphone apps significantly improve patient outcomes related to physical functional capacity during a CR program as opposed to lack of use of smartphone apps for cardiac outpatients who are using CR as a form of secondary prevention.

## Methods

### Search Strategy

A literature search was conducted through the University of Maryland’s Health Sciences and Human Services Library (HS/HSL) and ResearchGate. The following search terms were used: “[MeSH]” “smartphone applications”, OR [MeSH] “mobile app”, OR [MeSH] “mobile phone [MeSH] OR Smartphone apps, OR [MeSH] “digital health” AND [MeSH] “cardiac rehabilitation” [MeSH] OR “cardiovascular rehabilitation,” AND, “secondary prevention” AND “exercise”. The original article inclusion criteria were as follows: articles published between 2014 and 2020, and a study population of cardiac outpatients who suffered a cardiac event and who needed a CR program. The outcomes measured included exercise improvement during the 6MWT and PVO_2_. Peer-reviewed journal publications were included for completed RCTs in the English language. Due to the limited number of results, the search terms were expanded to include articles from 2014-2020 with the terms “mHealth” AND “mobile health” AND “telemonitoring” and to allow studies performed outside of the United States if they were compliant with AHA CR standards.

### Database Search Results

The search results from University of Maryland HS/HSL and PubMed included 27 articles, of which 8 reported on the wrong intervention, 6 focused on the wrong population or country, 2 measured the wrong outcomes, 4 consisted of abstracts only, 2 did not contain published results, and 2 were qualitative sources. This left 3 articles for the review. A search of ResearchGate found 16 articles, or which 1 was a duplicate, 6 focused on the wrong intervention, 2 focused on the wrong population or country, 3 measured wrong outcomes, 1 was qualitative, and 3 were used in this review. Therefore, a total of 6 articles were incorporated into this literature review. See Multimedia Appendix 1 for the PRISMA (Preferred Reporting Items for Systematic Reviews and Meta-analyses) diagram.

## Results

### Individual Evidence From RCTs

An unblinded RCT performed by Varnfield et al [11] tested the effectiveness of a smartphone app (or website for those without a smartphone) using biofeedback from the smartphone app to aid in obtaining automatic patient progress reports, recording, and goal setting during CR for patients who had experienced a past heart attack. For the duration of 6 weeks, followed by a 6-month maintenance period, both the control group (n=60), which included traditional in-center cardiac rehabilitation (TCR), and the at-home CR program with the smartphone app/internet, called the Care Assessment Platform of Cardiac Rehabilitation (CAP-CR) (n=60), completed components of the CR program, including exercise monitoring, educational information, motivational messages, and weekly mentoring appointments, to improve their cardiac health in order to prevent reoccurring cardiac events. The results showed that both groups had significantly improved 6MWT results (CAP-CR: 60 minutes, TCR: 47 minutes, *P*<.001), and the CAP-CR intervention group experienced significant weight loss (*P*=.02), experienced significantly better quality of life (baseline median score on the EuroQol-5D dimensions scale=.84 compared to .92 at 6 weeks, *P*<.001) and showed better adherence (94%) to CAP-CR compared to TCR (68%) (*P*<.05). See Table 1 for details.

Widmer et al [3] conducted a randomized single blind controlled trial to determine if TCR with the use of a digital health intervention, in the form of an application via a smartphone or website, would help decrease the readmission rates for hospitals and emergency departments compared to TCR with no digital health intervention. In the span of 180 days, 34 participants were tested in the control group and 37 were given treatment in the intervention group. Readmission rates were recorded along with secondary measurements such as weight, blood pressure, blood glucose, physical activity, diet, and quality of life. The digital health intervention encompassed diet, exercise, and education tasks for the patients to complete. The results showed that there was no significant change in readmission rates between TCR and rehabilitation with the addition of the smartphone app or website (*P*=.054). Also, the difference in exercise/walking ability was not significant (*P*=.35). However, between the two groups, the digital health intervention group saw a significant reduction in weight and body mass index (*P*=.02) compared to the TCR group.

Maddison et al [18] used a mobile phone intervention, Heart Exercise And Remote Technologies (HEART), to study the effects of delivering text messages and videos to patients at home to increase exercise capacity through encouragement and reminders for an at-home exercise program. Although this was a good theory in practice, and the study had a large sample size of 171 participants, the intervention alone was not strong enough to create significant results, and it was determined that exercise capacity in the form of PVO_2_ through respiratory gas analysis did not show significant changes during exercise before the program and after 24 weeks (*P*=.65).

In an 8-week-long study performed by Yudi et al [15], 168 acute coronary syndrome patients were tested for a program, of which 83 patients used a smartphone-based secondary prevention program with TCR compared to 85 patients using TCR alone. The smartphone app group had significant results for exercise capacity, as measured by the standard 6-minute walk test (*P*=.02). Additionally, compared to TCR alone, using a smartphone app facilitated program acceptance and mental well-being.

**Table 1:** Evidence summary.

Authors, Year	Objective	Evidence rating^a^	Design	Sample	Intervention	Outcome measurement	Result/recommendation
Lunde et al, 2020 [19]	Smartphone apps in CR^b^ completion and follow-up for one year compared to traditional CR with no apps	II	Single-blind RCT^c^	113 participants at the end of and after CR with n=54 in the control group (no app) and n=48 in the intervention group (app)	Smartphone apps used with/after CR compared to traditional CR with no app	PVO_2_^d^, goals achieved, new exercise habits, exercise ability, BP^e^, body weight, quality of life, lipid profile, triglycerides	Both traditional CR and CR with smartphone apps were significant in improving VO_2_, goal achievement, and exercise ability
Maddison et al, 2015 [18]	To test the effectiveness of a mobile CR home exercise program	II	Parallel two-arm RCT	New Zealand patients with IHD^f^ (N=171; control=86; intervention=85)	HEART^g^, a mobile phone program that delivers automatic personalized text messages to increase behavior and motivation for exercise	Exercise capacity measured by PVO_2_	Mobile phone program failed to increase exercise capacity in patients with IHD
Rosario et al, 2018 [16]	Smartphone app (STAHR^h^ app) used between CR sessions to increase the completion rate of CR and help improve clinical outcomes for patients	II	Unblinded RCT	Australian patients in need of CR (N=66; control=33; smartphone app with medical equipment=33)	Smartphone app capable of automatically recording data from blood pressure cuff and weight scale while completing CR compared to CR group without app	Completion of CR, 6MWT^i^, BP, heart rate, weight	Completion rates not significant between groups, but results for 6MWT were significant, and the intervention group improved significantly compared to the control group
Varnfield et al, 2014 [11]	To test smartphone app use and health impact during home CR	II	Unblinded RCT	Australian patients post-MI^j^ (N=120; intervention=60; TCR^k^=60)	Effect of comprehensive smartphone app in home (CAP-CR^l^) on CR outcomes and use compared to TCR with no smartphone app	Modifiable factors: 6MWT for functional capacity, survey of diet, BP, heart rate, BMI, waist circumference, and lipid test, as well as general acceptability, adherence, completion	Both groups indicated significant improvement in 6MWT (TCR: 47 minutes, CAP-CR 60 minutes) with CAP-CR improving weight loss, diet, and emotional state. Home CR program using smartphone apps can improve post-MI CR use with positive clinical results
Widmer et al, 2017 [3]	Use of a smartphone app (or same program on the web) during CR can decrease ED visits and hospitalization	II	Single-blind RCT	US PCI^m^ and ACS^n^ patients (N=71; CR and app=37; just CR [control]=34)	Smartphone app (or website with same features) during CR compared to CR with no app or website	Number of ED^o^ visits during study and number of walking minutes tolerated between the two groups	Overall failed to benefit patients, with no significant difference in exercise capacity or walking ability, but had significant weight loss and BMI improvement for patients. More studies should be conducted on larger scales.
Yudi et al, 2020 [15]	Use of a smartphone app intervention with traditional CR as secondary prevention for patients with ACS	II	Single-blind, two-arm, parallel RCT	New Zealand patients with ACS (N=168; control=85; smartphone app and TCR=83)	Smartphone app used with TCR compared to TCR alone	Exercise capacity by 6MWT	Results showed significant improvement for 6MWT with an increased distance in the smartphone app group, and the smartphone group was more likely to use CR. There was no difference for either group in smoking cessation.

^a^Evidence ratings for clinical studies: I=systematic review of randomized controlled trials, II=randomized controlled trial, III=quasi-experimental study not randomized, IV=qualitative study, V=systematic review of qualitative studies, VI=qualitative study, VII=expert opinion.

^b^CR: cardiac rehabilitation.

^c^RCT: randomized controlled trial.

^d^PVO_2_: peak oxygen uptake.

^e^BP: blood pressure.

^f^IHD: ischemic heart disease.

^g^HEART: Heart Exercise And Remote Technologies

^h^STAHR: Smartphone Technology and Heart Rehabilitation.

^i^6MWT: 6-minute walk test.

^j^MI: myocardial infarction.

^k^TCR: traditional in-center cardiac rehabilitation.

^l^CAP-CR: Care Assessment Platform of Cardiac Rehabilitation.

^m^PCI: percutaneous coronary intervention.

^n^ACS: acute coronary syndrome.

^o^ED: emergency department.

A study completed in 2018 by Rosario et al [16] took a novel approach of creating a smartphone app that could wirelessly connect to a blood pressure cuff and weight scale, so that when the health technologies were used, information would automatically be downloaded to the app. Using 66 participants in a CR program (33 in the control group), this adjunctive smartphone technology was used between in-patient CR sessions to help patients record health information and keep up with the CR requirements at home to encourage active participation and decrease dropout rates. Apart from completion rates measured, the other main outcome was a 6MWT, which helped determine if using the automatic built-in pedometer and smartphone health monitoring equipment could achieve clinically significant results in exercise capacity. The experiment was shown to have significant results for completion and participants’ exercise capabilities (*P*=.01).

A recent article, in 2020, by Lunde et al [19] focused on peak oxygen uptake and exercise ability in a maintenance period during and after CR, by way of a 1-year follow-up, of patients who used a smartphone app compared to TCR with no app. A single-blind RCT was performed with 113 participants, a control group (n=56) and an intervention group (n=57), with the intervention group receiving encouragement and personal goal-driven reminders on the app to complete CR activities a few times a week. The primary assessment, PVO_2_, was significant for both groups, with *P*=.001 for the intervention group and *P*=.002 for the control group. Secondary assessments of goal achievement, new exercise habits, and exercise ability were significant for both groups (intervention group: *P*=.013; control group: *P*=.014). This study recommends the use of smartphone apps in aiding patients with CR and for the prevention of secondary coronary events.

### Evidence Summary

Overall, from all the studies combined, the average age of participants was 57 years, with 536/709 males (75.6%) and 173/709 females (24.4%). Sample sizes varied from study to study, so caution should be used when applying these data to the entire cardiac outpatient population in need of CR. The number of study participants ranged from 6 to 171 [18], with a median number of 73 participants [3,16], 42 days [16] (with 6-month follow up) [11], 56 days [15], 168 days [18], 180 days [3], and 1 year [19].

Inclusion criteria for all study participants were as follows: received a referral for CR [11,16], English speaking [16,18,19], literate [18], clinically stable [16,18,19], age older than 18 years [15,16,19], and ownership of a smartphone [15,19]. Exclusion criteria were as follows: senses too impaired to use a smartphone [11], not owning a smartphone [15,18], terminal or unstable prognosis [15,18,19], and untreated ventricular tachycardia [15,19].

Table 2 provides a list of the interventions used in the smartphone CR programs.

Table 3 shows the main outcome measured, physical functional capacity either through the MWT or PVO_2_ uptake, as well as other secondary outcomes.

There have been mixed outcomes regarding the use of smartphone apps in CR for improving exercise functional capacity. Overall, the use of smartphone apps and their acceptance in CR is gaining traction, even among older patients [20]; however, clinical outcome results are inconsistent.

**Table 2:** Comparison of important variables.

		Lunde et al, 2020 [19]	Maddison et al, 2015 [18]	Rosario et al, 2018 [16]	Varnfield et al, 2014 [11]		Widmer et al, 2017 [3]		Yudi et al, 2020 [15]	
Usability/feasibility/utility			✓	✓	✓				✓	
Adherence		✓			✓				✓	
**Cardiac rehabilitation education**						✓		✓		✓
	Exercise/walking prompts		✓						✓	
	Medication support								✓	
	Encouragement	✓	✓						✓	
	Dietary help				✓		✓		✓	
Automatically sent data to physicians				✓						

**Table 3:** Exercise function capacity and contributing factors.

	Lunde et al, 2020 [19]	Maddison et al, 2015 [18]	Rosario et al, 2018 [16]	Varnfield et al, 2014 [11]	Widmer et al, 2017 [3]	Yudi et al, 2020 [15]
Exercise function capacity (6MWT^a^/compliance/ PVO_2_^b^)	**+** ^c^	–^d^	**+**	**+**	–	**+**
Change in blood pressure/heart rate				+		–
Weight loss					+	
Usability/feasibility		✓^e^	✓	✓		✓
Lipid profile				+	–	
Hospital readmission or death occurred	✓				✓	✓
Cardiac rehabilitation phase^f^	III	II/III	N/A^g^	N/A	II	I/II

^a^6MWT: 6-minute walk test.

^b^PVO_2_: peak oxygen uptake.

^c^+: significant improvement for intervention group.

^d^–: no significant improvement in intervention group.

^e^✓: measured.

^f^Phase I: in-patient phase; Phase II: patient begins outpatient rehabilitation and develops a comprehensive treatment plan; Phase III: maintenance phase.

^g^N/A: not applicable.

## Discussion

### Principal Findings

Currently, the results are mixed for studies on the use of smartphone apps in CR to improve physical functional capacity. However, a key observation that should be noted is that some of the distinguishing differences between clinically failed smartphone CR and improvements in patient outcomes were associated with apps that included an automaticity component for recording progress (such as an automatic step counter) [11-13], providing real-time feedback on progress, automatic logging of information, or correctional goal setting [11,12,16,17]. Conversely, the apps that were not as successful at creating clinical outcomes for exercise capacity were the apps that constantly required patients to record their data, placed the patients in CR too soon after the cardiac event, and focused on only one intervention aspect of CR [18].

CR smartphone apps that implement correctional feedback and/or automatic recording during exercise programs and portions of CR yielded positive results for increased exercise capacity and compliance [11,16]. A contributing factor in this finding may be that motivational level is often overlooked with these programs; patients want to get better, but sustaining motivation can be difficult with boring tasks, such as manually recording data every few hours. Additionally, being able to see one’s performance in real time is a motivating factor, as discovered by Varnfield et al [11] and Rosario et al [16], who had success with exercise compliance and improvement when patients could see their step count through the app’s accelerometer and the information was automatically logged. Rosario et al [16] found that the most accepted CR management component was the smartphone app’s near-field communication abilities (ie, downloading the blood pressure results and weight results automatically to the phone app as well as the built-in pedometer for recording steps).

The unsuccessful CR smartphone outcomes were obtained for the apps that heavily relied on self-reporting surveys and patient-recorded progress and were overall unable to increase the patient’s exercise capacity during CR [3,18]. Behavioral motivation is a substantial component of patients who use a CR program for secondary prevention of cardiac events. This is a difficult aspect to address, and although some articles, such as that by Maddison et al [18], did attempt to encourage use of a CR program by text messaging encouragement, this intervention alone is not strong enough to enable motivational behavior change. In addition, Rosario et al [16] reported that questionnaires that collected data were only completed by 22 out of 66 participants (33%), and this was the least successful intervention to keep participants engaged in CR. Finally, another good example of how self-reporting data and surveys create an ambivalent patient experience on improved results was reported by Vuorinen et al [21], who obtained unsuccessful results for decreasing myocardial infarction readmission rates. Their CR program and smartphone app did not specifically address any exercise component; however, they discovered that data collection via patient report in the app was inaccurate because many of the patients stopped recording results for interventions, such as blood pressure and medication adherence [21]. Patients had a tendency to falsify reports and felt anxious while constantly recording their results because it made them hyperaware of their heart condition. It was suggested that automatic data transfer be used to accommodate these issues.

Another factor to consider when patients participate in a CR program with a smartphone app is to evaluate what phase of CR they are performing, because the starting health and clinical stability of patients differs between phases. It has been noted that for Phase I of CR with smartphone apps, patients are more likely to have higher hospitalization rates, deaths, and cardiac exacerbations because they are less stable at the start of the program [13]. However, this is a sad paradox because the patients who need CR the most are the ones who are the sickest and least stable, and so it is suggested that further research and brainstorming should be aimed at creating alternatives to reach this population.

One demerit to the current body of research is that some of the sources had small sample sizes [3,11], which can skew data and lead to biased interpretations due to a nonrepresentative sample. Another drawback to using smartphone apps is that overall, they are poorly regulated and easily misguided. iTunes alone claims to offer 43,000 wellness apps, but many of these are mislabeled [22]. Moreover, of the 710 cardiac apps, only a few are intended for CR [22]. Therefore, the smartphone apps chosen for this review were consciously picked for their evidence-based approach related to CR.

Overall, there is a lack of evidence-based literature to support the notion that smartphone apps have clinical impact related to exercise in cardiac disease management via acting as, or with, a CR program compared to the traditional in-person rehabilitation or at-home CR with no app support. Although many articles suggest that there is potential for these apps, to date, the overwhelming focus has been on determining if there is interest in a smartphone app for CR rather than if it is clinically effective. Large-scale scientific testing in the United States is the next step, and there are numerous protocols suggesting that RCTs are in the process of being conducted; however, the results of these studies have yet to be published.

Another problem is that in the available research regarding completed RCTs, some of the current apps in telehealth focus on the exercise portion of CR and ignore the other important interventions set by the AACVPR and AHA, such as individual assessment, nutrition, management of blood pressure, lipids, diabetes, exercise education, psychosocial support, and medication compliance. Studies that only focused on one CR component did not show improved cardiac patient health [18]. To combat cardiac illnesses, a multitiered approach is recommended because the heart is a complicated organ. Therefore, it is appropriate for smartphone app interventions to include more than one component of CR. However, a drawback of this approach is that it is difficult to test and to determine the effects of individual interventions on a certain outcome due to the possibility of confounding variables.

### Conclusions

The quality and safety implications of using smartphone apps include the ability to monitor the health status of patients from a remote location [13], increased communication with professionals from the medical team [12,13], and increased motivation for patients to take control of their own health [12]. Additionally, in the health care setting, language barriers can often create miscommunications and hinder the level of care given. Smartphone apps can be presented to patients in multiple languages; therefore, better-quality care can be administered [12]. Currently, the research for using smartphone apps with CR is not strong enough for cohesive translation into practice. Suggestions can be made for future studies based on current trends. For example, it should be recommended that CR app developers keep the starting health of their patients in mind because the physical/mental ability to use an app determines compliance in app use [13]. Furthermore, better coordination between health care professionals and app developers should occur for content creation to ensure that the workflow and CR program improves patient health rather than hindering it. It has also been suggested that as advocates for CR, physicians can prescribe CR apps for patients in rural areas or when there are transportation difficulties. However, because there are numerous apps on the market, these apps should be researched further to ensure that they aid in achieving better patient outcomes [22]. The apps that had the most impact were the ones that used remote sensing technologies to monitor some aspects of the patients’ health and gave real-time feedback for appropriate goal setting related to the individual’s needs for their CR program [11]. More research is required on smartphone apps, but as technologies are quickly advancing and telehealth is becoming more prevalent, a new direction of research should also include analysis of newer technologies that pair with smartphone apps, such as watches, with biosensing capabilities that can now detect alarming arrhythmias [13,20].

A key finding from this literature review is that there was a positive correlation between automatic biosensing capabilities and feedback apps when used in a multi-factorial CR approach and the physical functional capacity of cardiac patients. These current trends in the literature suggest smartphone apps can be used to aid CR if the key CR components are used in conjunction with biosensing abilities. However, other components, such as simple texting, self-logging information, and unstable health prior to CR, are ineffective in supporting rehabilitation efforts.

## Appendix

Multimedia Appendix 1 PRISMA (Preferred Reporting Items for Systematic Reviews and Meta-analyses) flow diagram.

## Abbreviations

6MWT: 6-minute walk test
AACVPR: American Association of Cardiovascular and Pulmonary Rehabilitation
AHA: American Heart Association
CAP-CR: Care Assessment Platform of Cardiac Rehabilitation
CR: cardiac rehabilitation
HEART: Heart Exercise And Remote Technologies
HS/HSL: Health Sciences and Human Services Library
PRISMA: Preferred Reporting Items for Systematic Reviews and Meta-Analyses
PVO_2_: peak oxygen uptake
RCT: randomized controlled trials
TCR: traditional in-center cardiac rehabilitation
