# Supplementary material for: Use of Smartphone Apps for Improving Physical Function Capacity in Cardiac Patient Rehabilitation: Systematic Review
Source: JMIRx Med. 2021 Sep 17;2(3):e21906. doi: 10.2196/21906 (PMC10414376; doi:10.2196/21906)
Supplement: Multimedia Appendix 1 [file xmed_v2i3e21906_app1.doc]

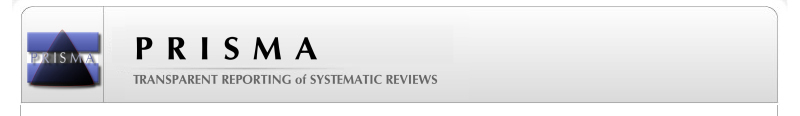
**PRISMA 2009 Flow Diagram**

**Screening**

**Included**

**Eligibility**

**Identification**

Records identified through University of Maryland HS/HSL and PubMed databases searches
(n = 27 )

Additional records identified through Research Gate
(n = 16)

Records after duplicates (n=1) removed
(n = 42)

Records screened
(n = 42)

Records excluded with reasons

- Wrong intervention (n=14)
- Wrong population/country (n=8)
- Wrong outcomes measured (n=5)
- Abstracts only (n=4)

(n = 31)

Full-text articles assessed for eligibility
(n = 11)

Full-text articles excluded, with reasons

- Did not have published results (n=2)
- Qualitative synthesis (n=3)

(n = 5)

Studies included in quantitative synthesis (meta-analysis)
(n = 6)
